# Evaluating a targeted educational intervention to promote high-value, cost-conscious care among medical students

**DOI:** 10.1080/10872981.2026.2673290

**Published:** 2026-05-13

**Authors:** Poojita Chinmay, Mohammed Khan, Zhidong Wang, Nivan Lakshman, Nicole Rockich-Winston

**Affiliations:** a Medical College of Georgia at Augusta University, Augusta, Georgia, USA; b Department of Pharmacology and Toxicology, Medical College of Georgia at Augusta University, Augusta, GA, USA

**Keywords:** High-value care, cost-conscious care, value-based care, medical education, curriculum development

## Abstract

Rising healthcare costs in the United States, with estimates suggesting up to 25% of spending is wasteful, have created an urgent need for cost-conscious, value-based care (VBC). VBC aims to improve patient outcomes relative to costs, yet its integration into medical education remains inconsistent, leaving future physicians underprepared for cost-sensitive practice. Early exposure to VBC principles can promote resource stewardship and reduce unnecessary interventions, yet few studies have rigorously evaluated structured educational interventions for medical students. This study examined the effectiveness of a single one-hour educational intervention on high-value, cost-conscious care (HVCCC) in improving pre-clerkship students' attitudes, confidence, and preparedness to apply VBC concepts in clinical practice. Pre-clerkship students (Class of 2027, Medical College of Georgia) completed pre- and post-lecture surveys using the validated Maastricht HVCCC Attitude Questionnaire (MHAQ), which measures three domains: Provision of High-Value Care, Integration of Costs, and Perceived Drawbacks. Additional items assessed self-efficacy and communication confidence regarding cost-related discussions. Paired *t*-tests compared pre- and post-intervention responses. Of the 85 students who participated, 78 provided paired responses for MHAQ items, while all 85 completed the additional non-MHAQ survey items. Following the lecture, total MHAQ scores increased significantly (mean 70.48 to 72.31; *p* < 0.001), indicating a more favorable overall attitude toward HVCCC. Both the Provision of High-Value Care and Integration of Costs domains improved significantly (*p* < 0.001 for each), while Perceived Drawbacks decreased (*p* = 0.019), suggesting reduced concerns about the negative consequences of HVCCC. Students also demonstrated gains in self-efficacy: confidence discussing cost-conscious care increased by 28–30 percentage points across communication settings, and 90.6% agreed they felt better equipped to initiate VBC conversations. A brief, structured HVCCC lecture improved medical students' attitudes, confidence, and preparedness for value-based practice. Integrating targeted VBC education early in training may foster sustainable, cost-conscious, and patient-centered care.

## Introduction

The United States spends nearly one-fifth of its gross domestic product on healthcare, yet underperforms on outcomes such as life expectancy and avoidable mortality [1]. An estimated 25–30% of spending is wasted due to unnecessary tests, procedures, redundancy, and administrative inefficiencies, while many Americans face medical debt. In response, high-value, cost-conscious care (HVCCC) has gained prominence. Physicians influence both the delivery and perception of care, yet formal training in value-based care (VBC) remains limited. Initiatives such as the American Board of Internal Medicine's *Choosing Wisely* campaign encourage clinicians to weigh costs alongside benefits and harms, aiming to minimize low-value practices without compromising patient care [2,3].

Despite these efforts, medical students often receive fragmented exposure to HVCCC, with limited reinforcement or integration into clinical reasoning. Professional identity and decision-making patterns develop early, suggesting that early and repeated exposure to HVCCC principles may be influential. While targeted educational interventions have demonstrated improvements in knowledge and awareness of VBC, evidence is less robust regarding their effects on attitudes, self-efficacy, and communication [4,5].

Educational approaches at this stage often emphasize foundational concepts, early clinical reasoning frameworks, and communication skills that can be reinforced longitudinally throughout training.

Prior studies have explored HVCCC and value-based care education across undergraduate and graduate medical training, with many interventions focusing on knowledge acquisition, clinical decision-making, or reducing unnecessary testing within clinical settings [4–6]. However, these efforts are often concentrated during clerkship or residency training, with less attention given to pre-clerkship learners, where foundational attitudes and clinical reasoning patterns begin to form. Additionally, while existing curricula have demonstrated improvements in knowledge, awareness, and selected competencies related to HVCCC, relatively fewer studies have evaluated changes in attitudes, perceived barriers, and communication self-efficacy—factors that may influence translation of knowledge into clinical practice [4–6]. There is also limited evidence evaluating brief, scalable interventions that can be integrated early in training without requiring extensive curricular restructuring.

To address these gaps, this study evaluated the impact of a structured, one-hour HVCCC educational intervention on pre-clerkship medical students' attitudes, perceived drawbacks, and confidence in applying value-based care principles. In particular, the study assessed shifts in students' confidence in initiating conversations about costs with patients and supervising physicians.

## Methods

This pre-post study was conducted at the Medical College of Georgia among pre-clerkship students (Class of 2027). All were invited to attend a single one-hour educational intervention divided into two components; survey participation was voluntary and anonymous. Analysis included only paired pre/post responses.

- Session 1: Principles of HVCCC; economic/clinical consequences of unnecessary utilization; strategies to reduce waste.
- Session 2: Communication skills for VBC conversations with residents/attendings, guidelines, and frameworks to discuss test appropriateness and treatment alternatives.

The single one-hour lecture was designed as a structured educational intervention aligned with the domains assessed by the Maastricht HVCCC Attitude Questionnaire (MHAQ) and the additional self-efficacy measures. Session 1 focused on foundational principles of HVCCC, including drivers of healthcare costs, identification of low-value care, and frameworks for evaluating the benefits, harms, and costs of clinical decisions, aligning with the domains of Provision of High-Value Care and Integration of Costs. Session 2 emphasized application of these principles through communication strategies for discussing cost-conscious care with patients and supervising physicians, as well as approaches to navigating hierarchical clinical environments. This session was designed to reinforce the application of HVCCC concepts and support the development of communication confidence and preparedness, as assessed in the additional survey items. Together, the sessions were intentionally structured to target attitudinal and behavioral constructs measured in the pre- and post-intervention surveys. Additional details regarding session structure, facilitators, and instructional materials are provided in the Supplementary Appendix.

Surveys were administered immediately before and after the sessions. The Maastricht HVCCC Attitudes Questionnaire (MHAQ) was used, covering three subscales: Provision of High-Value Care (8 items), Integration of Costs (10 items), and Perceived Drawbacks of HVCCC (7 items) [7]. Of the 29 items in the pre-lecture survey, 25 comprised the full set of items from the validated Maastricht HVCCC Attitudes Questionnaire (MHAQ) and were used without modification. The post-lecture survey included these same 25 items along with five additional questions designed to assess changes in students' self-efficacy, confidence, and preparedness in applying HVCCC concepts. Both surveys utilized a 4-point Likert scale (1 = strongly disagree, 4 = strongly agree).

Prior to administration, the survey was reviewed by faculty with expertise in medical education and health systems to assess clarity, relevance, and alignment with the educational objectives of the intervention. Minor revisions were made based on this feedback.

Surveys were administered in paper format immediately before the first session (pre-survey) and immediately following the second session (post-survey). Completed surveys were collected in person at the conclusion of each session. All responses were anonymous, and no identifying information was collected.

The survey was distributed to *N* = 85 students who attended the lecture. A total of *n* = 78 students completed both the pre- and post-intervention surveys for the MHAQ items (91.8% survey response rate), and a total of *n* = 85 students completed the pre- and post-intervention surveys for the non-MHAQ items (100% survey response rate). Only paired responses were included in the final analysis.

Statistical analyzes (JMP, SAS Institute Inc., Cary, NC) included descriptive statistics and paired t-tests comparing pre- and post-scores for total MHAQ score, subscales, and four non-MHAQ items present on both surveys. For the fifth post-only item, mean, standard deviation, and standard error were reported. Statistical significance was set at two-tailed *p* < 0.05.

Although Likert-scale responses are ordinal, aggregated MHAQ and subscale scores were treated as continuous variables, consistent with prior educational research using similar instruments. Given the sample size and use of composite scores, paired t-tests are considered appropriate and robust to modest deviations from normality.

### Ethical considerations

This study involved anonymous, voluntary survey data collected as part of a curricular educational activity and posed minimal risk to participants. The study was reviewed by the Medical College of Georgia Institutional Review Board and determined to be exempt from full IRB review prior to study commencement.

## Results

Seventy-eight students completed the pre- and post-intervention MHAQ surveys, and 85 students completed the non-MHAQ survey items. The total MHAQ score improved significantly from 70.48 to 72.31 (mean difference = 1.83 [95% CI: 0.91–2.75], *p* < 0.001). Across subscales, Provision of the high-value care subscale increased from a mean of 3.27 to 3.39 (mean difference = 0.12 [95% CI: 0.06–0.18], *p* < 0.001) and Integration of Costs subscale increased from 2.94 to 3.09 (mean difference = 0.15 [95% CI: 0.09–0.21], *p* < 0.001). Conversely, Perceived Drawbacks decreased from 2.13 to 2.03 (mean difference = –0.09 [95% CI: –0.17 to –0.01], *p* = 0.019), indicating a reduced perception of HVCCC as burdensome or harmful following the session.

**Figure 1.**
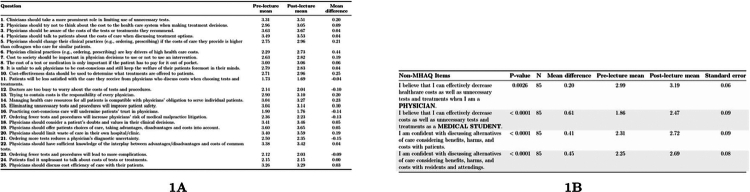
Summary of pre- and post-lecture survey responses evaluating medical students' value-based care attitudes and confidence. Panel 1A presents means and mean differences for all MHAQ items. Panel 1B summarizes paired t-test results for non-MHAQ items, demonstrating significant improvements across multiple domains.

At the item level (Figure 1A), Likert responses increased for 18 of 25 MHAQ items, decreased for six, and remained unchanged for one. The largest increases were observed for the following statements: ‘Physician clinical practices are key drivers of high health care costs’ (+0.44), ‘Cost-effectiveness data should be used to determine what treatments are offered to patients’ (+0.25).

Non-MHAQ items showed marked pre–post improvements in self-efficacy and communication confidence (Figure 1B). The proportion agreeing/strongly agreeing that they could decrease unnecessary costs *as future physicians* increased from 78.8% pre to 89.4%, and agreement that they could decrease costs *as medical students* rose from 17.7% to 50.6%. Confidence discussing alternatives (benefits, harms, and costs) *with patients* increased from 38.8% to 67.1% (+28.3 points), and *with residents/attendings* from 37.7% to 67.1% (+29.4 points). Consistent with these shifts, mean scores for the same four items also improved significantly (all *p* ≤ 0.0026). For the post-only item, 90.6% agreed or strongly agreed that the session left them better equipped to start value-based care conversations with other clinicians; the mean was 3.26 (SD 0.66, SE 0.07).

## Discussion

A structured HVCCC educational intervention improved students' attitudes toward value-based care and enhanced self-perceived confidence in discussing costs. Improvements were observed across attitudinal domains and in communication-related outcomes, suggesting that the intervention influenced how students approach cost-conscious decision-making. Non-MHAQ findings further indicate that students felt more prepared to initiate conversations about benefits, harms, and costs with both patients and supervising clinicians, a critical skill within hierarchical training environments.

Although the magnitude of change in total and subscale MHAQ scores was modest, this is consistent with prior HVCCC and value-based care educational interventions, which often demonstrate small but statistically significant shifts in attitudes following brief curricula [5,6]. The observed improvement in total MHAQ score corresponded to a small-to-moderate effect size (Cohen's d ≈ 0.44), suggesting that the intervention had a meaningful impact on student attitudes despite its brief format. In the context of pre-clerkship education, even modest changes may be meaningful, as attitudes and communication patterns are still forming and may influence future clinical decision-making. Rather than reflecting a limitation, these findings may represent an early signal of impact. Brief, targeted interventions producing measurable attitudinal and self-efficacy shifts suggest that HVCCC principles can be introduced effectively within limited curricular time. This is particularly relevant given the challenges of integrating new content into already dense pre-clerkship curricula. These early shifts may contribute to a foundation for reducing low-value practices over time, particularly when introduced early and reinforced longitudinally.

In addition, these findings align with prior studies demonstrating that HVCCC curricula can improve learner confidence and competencies [5,6]. Unlike many prior HVCCC educational interventions, which are implemented during clerkship or residency and often emphasize knowledge or utilization outcomes, this study focuses on pre-clerkship learners and targets attitudinal and communication-based constructs. Introducing HVCCC concepts at this earlier stage may help shape foundational clinical reasoning patterns and normalize cost-conscious decision-making before students enter clinical environments. Notably, this study demonstrates measurable effects from a concise, one-hour educational intervention with two components, supporting integration of HVCCC education into pre-clerkship curricula without requiring extensive curricular restructuring. Educational interventions may contribute to reducing low-value practices and waste while preserving patient safety [8]. Cultivating a value-based mindset early may support patient trust, reduce avoidable harm, and contribute to more sustainable healthcare delivery.

There are several limitations to consider. The study was conducted at a single institution, which may limit generalizability. The use of self-reported attitudes and confidence rather than observed behaviors introduces the possibility of response bias. Additionally, the use of immediate post-intervention assessments precludes evaluation of long-term retention or translation into clinical practice.

Future work should examine the durability of these effects, incorporate multi-institution cohorts, and evaluate the impact of longitudinal reinforcement strategies, such as case-based discussions, across the curriculum.

## Supplementary Material

Supplementary material Supplementary Appendix

## Data Availability

Data are not publicly available due to privacy and confidentiality considerations, but may be obtained from the authors upon reasonable request.

## Support

None reported.

## References

[1] Shrank WH, Rogstad TL, Parekh N. Waste in the US health care system: estimated costs and potential for savings. J Am Med Assoc. 2019;322(15):1501–1509. doi: 10.1001/jama.2019.13978 31589283

[2] Dreyer T, Joynt Maddox K. What's the value in value-based care? Washington, DC: AAMC; 2023. doi: 10.15766/rai_c7chwtb5

[3] Kassirer JP, Wong JB. Improving health outcomes and promoting stewardship of resources: the ABIM foundation’s choosing Wisely® campaign. AMA J Ethics. 2012;14(11):718–723. doi: 10.1001/virtualmentor.2012.14.11.fred1-1211 23351902

[4] El Fadel O, Goldberg ZN, Jain A, et al. Integrating choosing wisely, value-based care principles, into undergraduate medical education: a pilot study. Cureus. 2024 Mar 25;16(3):e56912. doi: 10.7759/cureus.56912 38528995 PMC10963070

[5] Pahwa AK, Eaton K, Apfel A, et al. Effect of a high value care curriculum on standardized patient exam in the core clerkship in internal Medicine. BMC Med Educ. 2020;20(1):365. doi: 10.1186/s12909-020-02303-1 33059679 PMC7560311

[6] Moriates C, Valencia V, Stamets S, et al. Using interactive learning modules to teach value-based health care to health professions trainees across the United States. Acad Med. 2019 Sep;94(9):1332–1336. doi: 10.1097/ACM.0000000000002670 31460928 PMC6727932

[7] Mordang SBR, Könings KD, Leep Hunderfund AN, et al. A new instrument to measure high value, cost-conscious care attitudes among healthcare stakeholders: development of the MHAQ. BMC Health Serv Res. 2020;20:156. doi: 10.1186/s12913-020-4979-z 32122356 PMC7053044

[8] Sadowski BW, Lane AB, Wood SM, et al. High-value, cost-conscious care: iterative systems-based interventions to reduce unnecessary laboratory testing. Am J Med. 2017;130(9):1112.e1–1112.e7. doi: 10.1016/j.amjmed.2017.02.029 28344140

