# Supplementary material for: Evaluating a targeted educational intervention to promote high-value, cost-conscious care among medical students
Source: Med Educ Online. 2026 May 13;31(1):2673290. doi: 10.1080/10872981.2026.2673290 (PMC13178031; doi:10.1080/10872981.2026.2673290)
Supplement: Supplementary material — Supplementary Appendix [file ZMEO_A_2673290_SM7888.docx]

**Supplementary Appendix: Educational Intervention Details**

**Structure and Duration**

The intervention consisted of a single one-hour session divided into two components. The first 30 minutes (Session 1) consisted of a didactic lecture introducing core principles of high-value, cost-conscious care (HVCCC). The second 30 minutes (Session 2) consisted of case-based learning designed to apply these principles in clinical scenarios.

**Facilitators**

The sessions were delivered by student representatives from the Costs of Care STARS (Students and Trainees Advocating for Resource Stewardship) program at the Medical College of Georgia, who had prior training and experience with HVCCC education.

**Instructional Content and Materials**

Content for both sessions was derived from the Costs of Care STARS national curriculum, which includes structured modules, toolkits, and interactive educational materials focused on value-based care and resource stewardship. This curriculum was adapted for delivery to pre-clerkship medical students.

**Interactive Components**

The first session was lecture-based. The second session incorporated case-based learning with interactive discussion, allowing students to apply HVCCC principles to clinical decision-making and practice communication strategies related to cost-conscious care.
